# Supplementary material for: Parameters estimation of gas capture through Mixed Matrix Membrane (MMM) with CFD
Source: PLoS One. 2025 May 13;20(5):e0322162. doi: 10.1371/journal.pone.0322162 (PMC12074613; doi:10.1371/journal.pone.0322162)
Supplement: Attachment — Submitted filename: Reviewe1.docx [file pone.0322162.s002.docx]

**Response to Reviewer Comments:**

We appreciate the reviewer’s comments to improve the manuscript. Reviewer comments are underlined, with our response below each comment. All modifications in the manuscript were marked in red color.

**Reviewer #1:** This paper is well-written. Just need to add some information.
(1) The authors should expand the Introduction section to better identify research gaps in current literature and emphasize the significance of this work.

- We appreciate the reviewer’s comments in the manuscript and highlighted in red.

(2) Please specify the software versions used in this study (e.g., MATLAB Academic 2023 or other relevant software).

- The manuscript has updated all the software versions used.

(3) Please provide more detailed information about the Artificial Neural Network (ANN) implementation, including:
• The dataset distribution (specify the number of samples used for training and validation)
• The network architecture (detail the number and configuration of layers)

- We explain more detailed information about the implementation of the artificial neural network (ANN). Updated the manuscript and highlighted in red

(4) Enhance the Results and Discussion section by incorporating more references to relevant literature and comparing your findings with previous studies.

-We appreciate the reviewer comments, the results were discussed and researchers in the same field were added.

Reviewer #2:

The manuscript titled "Parameters Estimation of Gas Capture Through Mixed Matrix Membrane (MMM) with CFD" investigates the potential of mixed matrix membranes (MMM) to capture carbon dioxide (CO₂) from natural gas using computational fluid dynamics (CFD). A 3D model was developed using COMSOL 6.1, combined with MATLAB's artificial neural network (ANN), to estimate critical parameters such as permeance and diffusion coefficients. The authors evaluated the impact of operating parameters such as feed pressure, temperature, and CO₂ concentration on the membrane’s performance. The work concludes that CFD modeling provides accurate predictions of gas separation performance for MMMs, while temperature exhibited minimal influence on separation efficiency.

Recommendation
The manuscript presents an important contribution to gas separation technologies, showcasing the integration of CFD and ANN in parameter estimation for MMMs. However, revisions are required to enhance the depth of analysis, methodological clarity, and overall presentation. I recommend major revisions to address the highlighted issues and improve the scientific rigor of the paper.

My comments are the following:

1. Expand on Novelty and Relevance: While the manuscript presents an innovative approach, the introduction could elaborate further on recent advancements in CO₂ capture using MMMs. Highlighting the novelty and the significance of the CFD-ANN integration would strengthen the impact of the study.

- Thanks for your comments. Your comments have been immensely helpful. We updated the paper

2. Enhance Methodological Clarity: Although the methods section mentions geometric models and computational tools, the description is somewhat brief. It is recommended to:
2.1 Provide more detail on the construction of the geometrical model used in the simulations.
2.2 Explain the rationale for selecting COMSOL 6.1 and MATLAB’s ANN for parameter estimation, particularly the advantages these tools offer over alternatives.

- Thanks for your advice, we have changed and updated the paper.

3. Validation and Comparative Analysis: The manuscript validates its model against one prior study, which limits confidence in the model's robustness. To address this:
3.1 Include comparisons with experimental or additional literature data to confirm the model’s reliability.
3.2 Provide a comparative analysis of MMM performance against other CO₂ capture methods (e.g., chemical absorption or alternative membranes). Tables or charts could enhance the clarity of these comparisons.

- We have revised and updated the paper based on your suggestions.

4. Analysis of Temperature Effects: The results section notes that temperature has minimal impact on separation performance, but the discussion is limited. A deeper analysis of how solubility and diffusivity counteract at varying temperatures would be beneficial.

- "We greatly value your interest, as we have provided an in-depth explanation along with detailed scientific insights."

5. Quantitative Metrics: The paper evaluates permeance and diffusion coefficients but lacks a broader context. Discuss how these metrics translate to industrial applicability, operational efficiency, and cost-effectiveness.

- We greatly appreciate this consideration as it was mentioned at the beginning of the "

3.4. Parameter Estimation"

6. Expand Future Directions: The conclusion briefly mentions potential future work. To provide clearer guidance for subsequent studies, consider:
6.1 Identifying specific challenges for scaling up the MMM technology.
6.2 Suggesting experimental validations or additional computational studies for further refinement.

- We appreciate the reviewer's comments. Added in the last paragraph of the conclusion There are challenges and future work that need to be addressed.

7. Ethics and Data Transparency: The manuscript does not mention ethical considerations or data availability. Even if ethics approvals are not required, this should be explicitly stated. Additionally, ensure all data complies with PLOS’s open data policy.

- We will change the sending method.

Minor Comments to follow to Enhance the Manuscript Overall Structure:

1- Correct typographical issues such as "Simlution" in Section 4.1 and ensure consistent use of terminology throughout the manuscript. Done

2- Improve figure captions to provide standalone clarity, including explanations for abbreviations and key observations. Done

3- Specify units for all parameters in equations to enhance reader comprehension.

Done
4- Clarify ambiguous statements such as "precise parameter estimates" by providing accuracy thresholds or numerical ranges. Done

5- Include the rationale behind the chosen experimental conditions (e.g., pressure and CO₂ concentration) in the methodology. Done

6- Provide interpretations of figures directly in captions to help readers quickly grasp their significance. Done

- We appreciate the reviewer’s comments to improve the manuscript. Reviewer comments are underlined, with our response below each comment. All modifications in the manuscript were marked in red color.

Thank you again for reviewing our manuscript and for your valuable comments. We appreciate your suggestions and advice. We look forward to hearing from you soon.

Sincerely,

Ali A. Abdulabbas

Corresponding author

che.20.02@grad.uotechnology.edu.iq
