# Supplementary material for: Parameters estimation of gas capture through Mixed Matrix Membrane (MMM) with CFD
Source: PLoS One. 2025 May 13;20(5):e0322162. doi: 10.1371/journal.pone.0322162 (PMC12074613; doi:10.1371/journal.pone.0322162)
Supplement: Attachment — Submitted filename: Revised Reviewer R2.docx [file pone.0322162.s003.docx]

**Response to Comments:**

1- In the abstract, authors should give a numerical value or percentage instead of precise parameter estimates.

- Thank you for your comment. According to your comment, a percentage has been added.

2- The second-to-last paragraph of the introduction section needs a well-cited reference.

-Thank you for your comment. According to your comment, references were added.

3- The formatting of the table needs to be checked. For example, in Table 9, there are no borders.

Thank you for your comment. Borders have been added to the table.

4- There are spacing issues between text and reference numbers

Thanks for your comment. Based on your comment, we have made an edit.

Thank you again for reviewing our manuscript and for your valuable comments. We appreciate your suggestions and advice. We look forward to hearing from you soon.

Sincerely,

Ali A. Abdulabbas

Corresponding author

che.20.02@grad.uotechnology.edu.iq
